# Genome-Wide Detection of Key Genes and Epigenetic Markers for Chicken Fatty Liver

**DOI:** 10.3390/ijms21051800

**Published:** 2020-03-05

**Authors:** Xiaodong Tan, Ranran Liu, Siyuan Xing, Yonghong Zhang, Qinghe Li, Maiqing Zheng, Guiping Zhao, Jie Wen

**Affiliations:** 1 Institute of Animal Sciences, Chinese Academy of Agricultural Sciences, Beijing 100193, China; tanxiaodong08@163.com (X.T.); liuranran112@126.com (R.L.); tcsxingsy@126.com (S.X.); yonghong@jlu.edu.cn (Y.Z.); qli2014@126.com (Q.L.); zhengmaiqing@caas.cn (M.Z.); 2 State Key Laboratory of Animal Nutrition, Beijing 100193, China; 3 Animal Breeding and Genomics, Wageningen University & Research, 6708 PB Wageningen, The Netherlands

**Keywords:** chicken, fatty liver, lipid metabolism, epigenetics, DNA methylation, long noncoding RNA

## Abstract

Chickens are one of the most important sources of meat worldwide, and the occurrence of fatty liver syndrome (FLS) is closely related to production efficiency. However, the potential mechanism of FLS remains poorly understood. An integrated analysis of data from whole-genome bisulfite sequencing and long noncoding RNA (lncRNA) sequencing was conducted. A total of 1177 differentially expressed genes (DEGs) and 1442 differentially methylated genes (DMGs) were found. There were 72% of 83 lipid- and glucose-related genes upregulated; 81% of 150 immune-related genes were downregulated in fatty livers. Part of those genes was within differentially methylated regions (DMRs). Besides, sixty-seven lncRNAs were identified differentially expressed and divided into 13 clusters based on their expression pattern. Some lipid- and glucose-related lncRNAs (e.g., LNC_006756, LNC_012355, and LNC_005024) and immune-related lncRNAs (e.g., LNC_010111, LNC_010862, and LNC_001272) were found through a co-expression network and functional annotation. From the expression and epigenetic profiles, 23 target genes (e.g., *HAO1*, *ABCD3*, and *BLMH*) were found to be hub genes that were regulated by both methylation and lncRNAs. We have provided comprehensive epigenetic and transcriptomic profiles on FLS in chicken, and the identification of key genes and epigenetic markers will expand our understanding of the molecular mechanism of chicken FLS.

## 1. Introduction

Chickens are one of the most important sources of meat worldwide and comprise 32.63% of all meat consumption, with more than 66.6 billion meat-type chickens produced in the world in 2017 [1]. With the experience of decades of breeding, the growth rate of chicken has been greatly improved. Nevertheless, excessive fat accretion is a crucial problem during the production, which could result in low feed conversion ratio, high cost of chicken production, as well as the excessive pollution to the environment.

For chickens, the liver is the core organ for lipid synthesis [2,3]. Lipid homeostasis is closely dependent on some hepatic metabolic pathways, including lipid absorption, lipid synthesis, β-oxidation, and lipoprotein transport, but the disorder of these pathways could lead to the fatty liver [4,5]. Fatty liver syndrome (FLS) is characterized by increased lipid accumulation in the liver, which is different from fatty liver hemorrhagic syndrome (FLHS) [6]. Both the mortality rate and egg production are quite different between the two syndromes [7,8]. But if chickens with FLS do not receive timely treatment, the birds will develop FLHS.

In the standardized farming of chickens, FLS is an inevitable problem, and it was reported that this incidence reached 4% or even close to 20% [9,10]. The study of FLS is difficult because of its obscure features and irregular occurrence. Usually, nutrients supplement is widely applied for the prevention of FLS, such as choline [11], betaine [12], polyunsaturated fatty acid [13], and conjugated linoleic acid [14]. However, this approach is costly and not precise enough. Therefore, the research on the molecular mechanism of FLS may be a better solution.

The environment can interplay with genetics in such a manner that traits can be modified [15,16]. This form of trait modification is influenced by epigenetic factors that mediate the effect of the environment on genetics. An example of the epigenetic modification is the biochemical modification of DNA by cytosine methylation of CpG dinucleotides. Variants in hepatic DNA methylation have been linked to lipid metabolism and fatty liver [17,18]. Hepatic methylation of *PPARγ* is higher in non-alcoholic fatty liver disease (NAFLD) subjects and correlated with plasma fasting insulin levels [19]. Site-specific changes in the methylation level of *FAS* have been found in male mice fed a high-fat diet [20]. Such results suggest that aberrant methylation of genomic DNA is an epigenetic modification that may relate to abnormal transcription in animals with fatty liver.

The importance of long noncoding RNA (lncRNA) has become a focus of hepatic lipid metabolism and fatty liver [21,22]. A large number of lncRNAs have been identified in the livers of NAFLD patients [23]. *FLRL8*, *FLRL3*, and *FLRL7* have been demonstrated to show an underlying effect on the Peroxisome Proliferators-activated Receptors (PPAR) signaling pathway by interaction with candidate genes related to lipogenesis and lipid transport, such as *FABP5*, *LPL*, and *FADS2* [22]. There are few investigations in animals with the one exception that Li et al. has identified, a metabolism-related lncRNA (lncLTR), which is regulated by estrogen and associated with plasma triglyceride in hens [24].

Both DNA methylation and lncRNA are closed related to fatty liver, the analysis of which is a valid approach to explore the molecular mechanism. For chickens under the standardized environment, considering that the FLS is inevitable and nutrition supplement is not precise enough, the research based on the molecular mechanism is urgently needed. Therefore, we started this investigation to detect the key genes and epigenetic markers associated with chicken FLS. Notably, all chickens were reared under the same environment. Whole-genome bisulfite sequencing and lncRNA/mRNA sequencing were performed, and the epigenetic and expression profiles were used to provide the new research targets, which could further the understanding of the molecular mechanism of chicken FLS.

## 2. Results

### 2.1. The Slaughter Performance and Serum Biochemical Indices of Chickens with Fatty Liver

**Figure 1:**
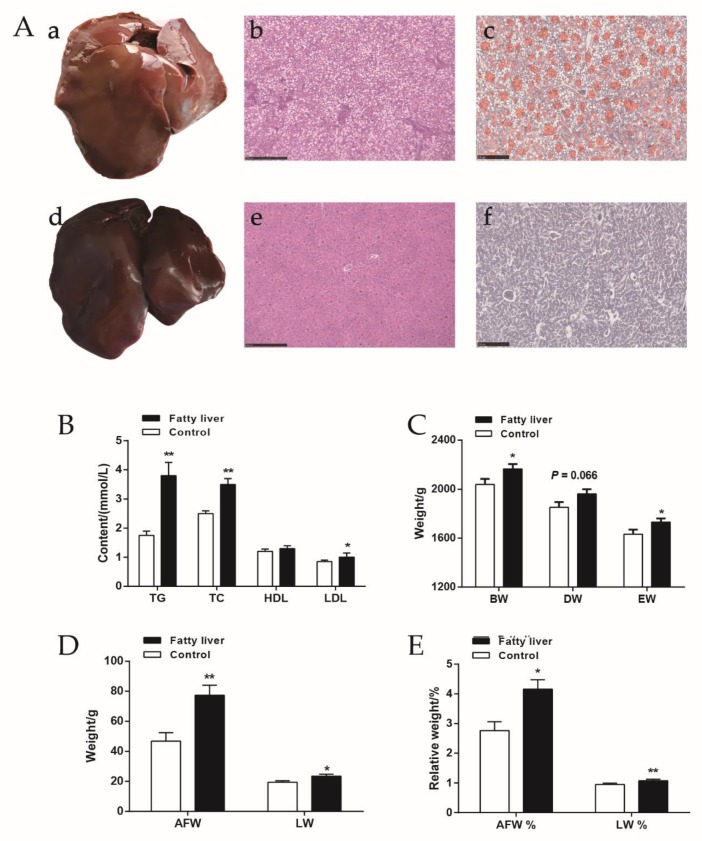
Liver histopathology and phenotypic identification. (**A**) The plots of liver histopathology. The plots of a–c represent the phenotype, HE staining, and Oil red O staining of fatty liver, respectively, while the plots of d–f represent the same items of normal liver (250 µm). (**B**) Contents of lipids in serum. Includes high-density lipoprotein (HDL), low-density lipoprotein (LDL), total cholesterol (TC) and triglyceride (TG), *n* > 10 per group. (**C**) Bodyweight (BW), dressed weight (DW) and eviscerated weight (EW) of chickens in two groups, *n* > 20 per group. (**D**) Abdominal fat weight (AFW) and liver weight (LW) of chickens in two groups, *n* > 20 per group. E Relative weight of abdominal fat (AFW %) and liver (LW %) of chickens in two groups, *n* > 20 per group. * represents *p* < 0.05, ** represents *p* < 0.01.

To assess the occurrence of fatty liver, we examined the extent of fatty degeneration by H&E and Oil Red O staining. Typical liver features are shown in Figure 1A with distinct pathological changes in chickens with fatty liver. Histological analysis indicated that more than 1/3 of hepatocytes had steatosis and massive accumulations of fat droplets within hepatocytes, as well as enlarged hepatocytes and abnormal liver leaflets (Figure 1A(a–c)). No signs of these abnormalities were observed in normal chickens (Figure 1A(d–f)).

The slaughter performance and serum biochemical indices are shown in Figure 1B–E. We tested the serum lipid-related index and found the content of triglyceride (TG), total cholesterol (TC), and low-density lipoprotein (LDL) to be significantly higher in the fatty liver group, while the serum high-density lipoprotein (HDL) content did not differ between the two groups (Figure 1B). For slaughter performance, body weight (BW) and eviscerated weight (EW) were significantly higher in the fatty liver group. The dressed weight (DW) had a similar trend between groups (*p* = 0.066, Figure 1C). The weight and the relative weight of abdominal fat and liver increased significantly in the fatty liver group of chickens (Figure 1D,E).

### 2.2. Transcriptome Profiling Analysis of Liver

**Figure 2:**
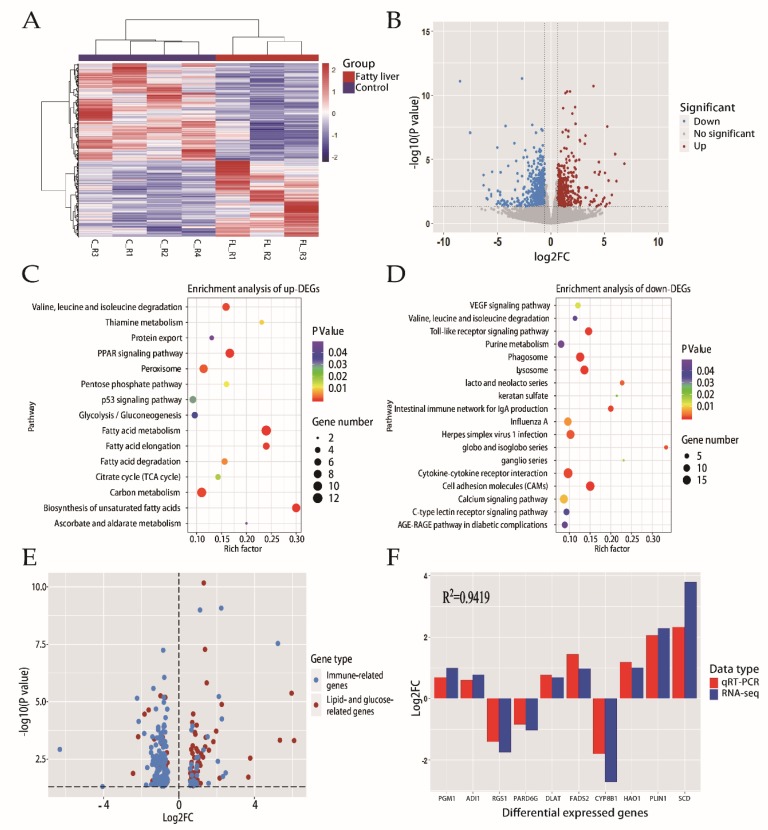
Transcriptomic changes and enrichment analysis in normal liver and fatty liver. (**A**) Heatmap of differentially expressed genes (DEGs) via hierarchical cluster analysis. Different rows correspond to different genes, red and blue strips represent up- and downregulation, respectively. (**B**) Volcano plot revealing DEGs with various *p*-values and fold changes (FC). Vertical line, FC = 1.5; horizontal line, *p*-value = 0.05. Red points, upregulated genes; blue points, downregulated genes. (**C**) Pathways of enrichment analysis with upregulated DEGs. Rich_factor, the ratio of imputed genes to background genes. (**D**) Pathways of enrichment analysis with downregulated DEGs. (**E**) The expression profile of lipid- and glucose-related DEGs and immune-related DEGs. (**F**) Verification results of qPCR for 10 randomly selected DEGs.

Hepatic gene expression profiles were assessed by RNA-seq for both the fatty liver and control groups (Figure 2A). We identified 1177 differentially expressed genes (DEGs), including 516 upregulated and 661 downregulated genes that differed between the fatty liver and control groups (Figure 2B, Table S1). Then we focused on the KEGG enrichment analysis with upregulated and downregulated genes, respectively.

For the 516 upregulated genes, 15 pathways were identified. Eight of which were lipid- and glucose-related pathways, including the Tricarboxylic Acid Cycle (TCA) cycle, biosynthesis of unsaturated fatty acids, fatty acid metabolism, the PPAR signaling pathway, and fatty acid elongation (Figure 2C). For the 661 downregulated genes, 18 pathways were predicted (Figure 2D). Most of these pathways were related to immune function, including cell adhesion molecules (CAMs), the toll-like receptor signaling pathway, and phagosome.

With special interests in DEGs involved in lipid- and glucose-related pathways and immune-related pathways. Sixty of 83 DEGs (72%) were upregulated and annotated to lipid and glucose metabolism. One hundred and twenty-one out of 150 DEGs (81%) were downregulated and annotated to immune function (Figure 2E).

RNA-seq results were validated by quantitative real-time PCR (qRT-PCR) analysis of 10 randomly selected DEGs (*PGM1*, *ADI1*, *RGS1*, *PARD6G*, *DLAT*, *FADS2*, *CYP8B1*, *HAO1*, *PLIN1*, *SCD*). Correlation analysis of expression results between qRT-PCR and RNA-seq showed a high relevance (R^2^ = 0.9419, Figure 2F), confirming the reliability of the RNA-seq data.

### 2.3. Integration Analysis of Methylome and Transcriptome

**Figure 3:**
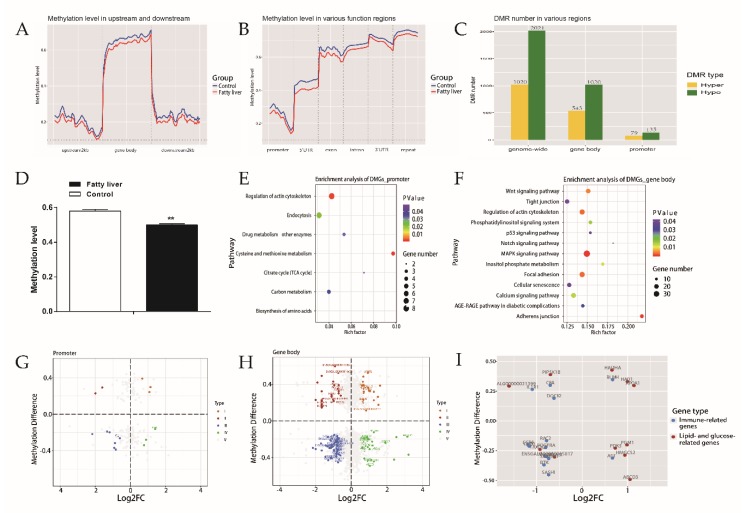
Methylation profile and enrichment analysis in normal liver and fatty liver. (**A**) Distribution of methylation in the gene body, upstream and downstream. Gene body, from transcription start site (TSS) to transcription end site (TES); upstream2k, two thousand base pairs of the upstream region from TSS; downstream2k, two thousand base pairs of the downstream region from TES. (**B**) Distribution of methylation in various regions, including promoter, 5′UTR, 3′UTR, intron, exon, and repeat region. (**C**) Statistics for differentially methylated region (DMR) number in the genome-wide range, gene body and promoter region. Hyper means the methylation level of DMR in the fatty liver group is higher than that in the control group, while hypo means the methylation level of DMR in the fatty liver group is lower than that in the control group. (**D**) Difference level of DMR overlapping with genes. ** represents *p*-value < 0.01. (**E**) Pathways of enrichment analysis with DMGs in the promoter. Rich_factor, the ratio of imputed genes to background genes. (**F**) Pathways of enrichment analysis with DMGs in the gene body. (**G**) Methylation and expression level of common genes with DMR in the promoter region. (**H**) Methylation and expression level of common genes with DMR in the gene body region. Type I: differential methylation (DM) > 0, log_2_FC > 0.585; type II: DM > 0, log_2_FC < −0.585; type III: DM < 0, log_2_FC < −0.585; type IV: DM < 0, log_2_FC > 0.585; type V: DMGs with no significant difference of expression level. (**I**) Methylation and expression level of lipid- and glucose-related genes and immune-related genes.

Overt differences in whole-genome DNA methylation were found between chickens with (*n* = 3) and without (*n* = 4) fatty liver. In comparison to control chickens, hepatic methylation levels of fatty liver chickens were lower for up- and downstream of the gene body and various functional regions (Figure 3A,B).

We identified a total of 3041 differentially methylated regions (DMRs) and 1442 differentially methylated genes (DMGs) by comparison of fatty liver and control groups (Table S2). Compared to the control group, the number of hypo-methylated (hypo) DMRs was greater in the fatty liver group (Figure 3C), while the methylation levels of DMRs overlapping with various gene regions were lower in the fatty liver group (*p* < 0.01, Figure 3D).

DMGs in the promoter were identified, including 80 hyper-methylated (hyper) genes and 139 hypo-methylated genes. KEGG enrichment analysis of those DMGs showed that seven pathways were significantly enriched (Figure 3E). Of the seven pathways, two (carbon metabolism and TCA cycle) were enriched by both DEGs and DMGs (Table 1).

DMGs in gene body regions were found, including 496 hypermethylated genes and 889 hypomethylated genes. Thirteen pathways were significantly enriched with those DMGs (Figure 3F). Three of the 13 pathways (calcium signaling pathway, p53 signaling pathway, and AGE-RAGE signaling pathway in diabetic complications) were also enriched by DEGs (Table 1).

A total of 127 genes were identified as both DEGs and DMGs (Table S3). By correlation analysis, it was found to be significantly associated between expression and methylation of 35 genes (*p* < 0.05), while the correlation of 17 genes between expression and methylation did not reach the statistical significance (*p* < 0.1) (Figure 3G,H). For DMRs overlapped with lipid- and glucose-related DEGs and immune-related DEGs, 12 were found in 11 lipid- and glucose-related DEGs (e.g., *HAO1*, *PDK3*, and *ABCD3*), and 16 were found in 14 immune-related DEGs (e.g., *BLMH*, *MARCH1*, and *CD80*) (Figure 3I), for example, immune-related gene *MARCH1* had hyper DMRs, which were significantly associated with gene expression. The lipid-related gene *ARNTL* had a similar regulatory relationship, and *ABCD3* may also have been affected by DNA methylation.

### 2.4. Integration Analysis of the LncRNA and the mRNA Profiles

**Figure 4:**
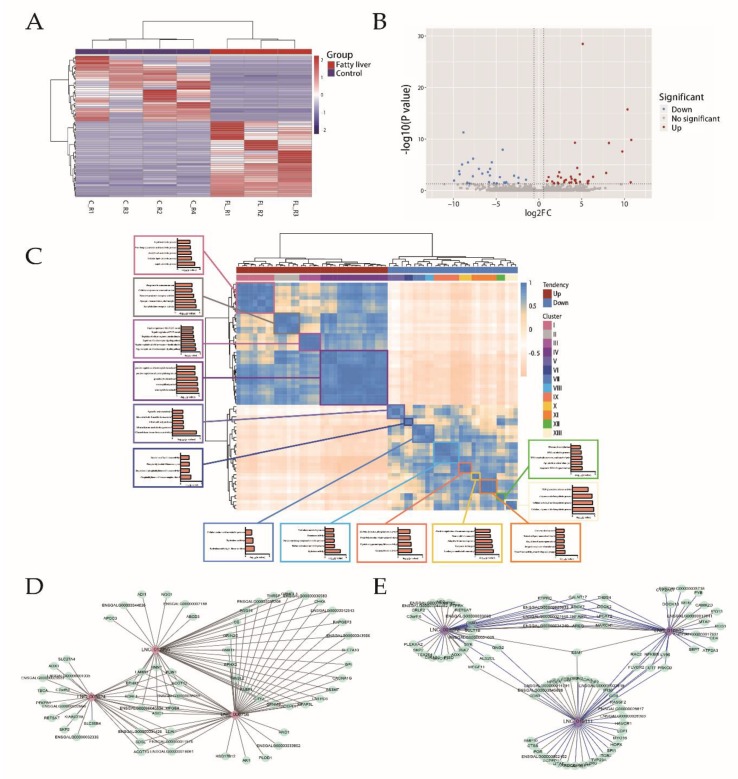
Expression profile of lncRNAs and interaction between lncRNAs and DEGs. (**A**) Heatmap of differentially expressed lncRNAs via hierarchical cluster analysis. Red and blue strips represent up- and downregulation, respectively. (**B**) Volcano plot revealing differentially expressed lncRNAs with various adjusted *p*-values and FC. Vertical line, FC = 1.5; horizontal line, adjusted *p*-value = 0.05. Red points, upregulated lncRNAs; blue points, downregulated lncRNAs. (**C**) Gene Ontology (GO) annotation with target DEGs of lncRNAs from each cluster. Boxes with various colors represent 13 clusters of lncRNAs. (**D**) Co-expression network between DEGs and lipid-related long noncoding RNAs (lncRNAs). The diamonds with various colors represent different clusters of lncRNAs, the circles with light green represent co-expression DEGs; grey line means the positive correlation between lncRNAs and DEGs, while blue line means the negative correlation between lncRNAs and DEGs. (**E**) Co-expression network between DEGs and immune-related lncRNAs.

The striking difference in global lncRNA expression profiles was found between the fatty liver and control groups (Figure 4A). Sixty-seven DE lncRNAs were identified (Figure 4B, Table S4), and those lncRNAs were divided into thirteen clusters by Pearson analysis. Potential *cis* and *trans* target genes were predicted and 56 differentially expressed *cis* target genes and 544 *trans* target genes were detected.

Correlations were sought between thirteen clusters of lncRNAs and DEGs. A set of 1290 co-expressed lncRNA/mRNA pairs and 544 *trans* target genes were identified (Table S5), from which a co-expressed network was constructed (Figure S1). Within the network, we found LNC_006756, LNC_001439, LNC_010098, LNC_010111, and LNC_001531 to have the highest degree, suggesting they may play a central role in the process of chicken FLS.

The putative biological function of lncRNAs was predicted by Gene Ontology (GO) enrichment analysis with co-expression DEGs by each lncRNA cluster (Figure 4C). We found that DEGs related to cluster I were associated with lipid metabolism. And LNC_006756, LNC_012355, and LNC_005024 were linked to many lipid metabolism-related genes, including *FABP1*, *PLIN1*, *MMP1*, *EPHX2*, *SLC27A4,* and *HAO1* (*r* > 0.95) (Figure 4D). Furthermore, DEGs related to cluster IV and X were associated with immune function. LNC_010111, LNC_010862, and LNC_001272 were found to be linked to immune-related genes, such as *DOCK2*, *MARCH1*, *PTPRC*, and *CD4* (Figure 4E). These results indicated that abnormal expression of lncRNAs had a major effect on the gene regulation underlying fatty liver development.

Chromosomal co-expressed genes within 300 kbs upstream and downstream of differentially expressed lncRNAs were assessed as potential *cis* genes. Fifty-nine lncRNAs were found to be cis-acting on 545 target genes, only 56 of which were differentially expressed between the two groups (Table S6). LNC_008671, LNC_009039, and LNC_010494 were found to have over 10 differentially expressed *cis* target genes.

### 2.5. Integration Analysis of the Methylation, the LncRNA, and the mRNA Profiles

By integration of DMR and lncRNA profiles, a list of DMRs was identified that overlapped with the genomic positions of 564 lncRNAs (Table S7). The methylation differences of most lncRNAs were between −0.5 and 0.5. Of those, the expression of five lncRNAs was significantly different between the two groups (Figure S2).

DMGs and targets of lncRNA regulated in the *cis* and *trans* way were overlapped to explore the candidate genes, which were related to both DNA methylation and lncRNAs. A total of 23 target genes were detected, including lipid- and glucose-related genes and immune-related genes (e.g., *HAO1*, *ABCD3*, and *BLMH*) (Table 2). Most target genes were associated with more than one lncRNA, with methylation differences mainly distributed in the gene body region.

## 3. Discussion

A high-fat diet (HFD) is a common and valid means by which to induce a fatty liver model. For the chicken line used in this study, only the Jingxing–Huang (JXH) chickens of the first generation (F0) were induced with HFD and offsprings were fed a normal diet. From the F1 to F3 generations, the incidence of FLS in the fatty liver group was twice as high as that in the control group [10]. No obvious genetic differentiation was observed between the two groups. As in mammals, offspring from parents with metabolic syndrome present a higher risk of metabolic abnormality [25,26]. Herein, the key genes and epigenetic markers for fatty liver may contribute to diagnosing FLS, and perhaps to explain the transgenerational effect of chicken FLS.

DEGs between chickens with and without fatty liver were assessed. Upregulated DEGs were mainly enriched in glucose and lipid metabolism pathways. The process of hepatic lipid synthesis was increased in the fatty liver group, with fat deposition rapidly increased [27]. We also found that downregulated DEGs were mainly enriched in immune pathways, such as the toll-like receptor signaling pathway. Previous studies showed female *TLR4*^−/−^ mice to have increased obesity but to be partially protected against HFD induced insulin resistance, possibly owing to reduced expression of inflammatory genes in the liver. The downregulated expression of *TLRs* in the liver may also increase obesity and play a crucial role in the occurrence of fatty liver. Gluckman et al. and Bruce et al. have suggested that DNA methylation modification is associated with fatty liver [17,18]. 

Exploring epigenetic modification with relation to gene expression has provided a new model by which to associate a genomic function to biological phenotypes and metabolism processes [17,18,28]. Previous studies have shown methylation changes to candidate genes to be related to NAFLD [29]. The integrated analysis herein permitted the detection of genome-wide changes in DNA methylation and in gene expression in chicken fatty liver.

DNA methylation in the promoter is negatively correlated with transcription, while DNA methylation of the gene body can affect transcription either positively or negatively. Jung et al. suggested that overexpression of *PDK3* promoted elevated levels of glucose aerobic oxidation, which has an important effect on liver disease [30,31]. In this study, this gene had higher expression and intron decreasing methylation in chickens with fatty liver. Reduced expression of *ARNTL* has been observed in those with obesity [32]. The gene is involved in the process of fat deposition with high levels of circulating fatty acids in the liver [33], which indicates *ARNTL* has a possible regulatory role in the process of hepatic lipid accumulation. Two bidirectional DMRs within *ARNTL* were significantly associated with reduced expression levels, which is consistent with previous reports. Elevated TCA cycle flux is often observed with fatty liver [34,35], but an epigenetic association with genes of the TCA cycle has not been reported. TCA cycle is the ultimate pathway of nutrient metabolism; therefore, our results provide a possible mechanism by which nutrients relieve chicken fatty liver syndrome. In this study, many other key genes (e.g., *IGF2BP1*, *ADI1*, and *HADHA*) were also detected, but the function of these genes has not been reported to relate to fatty liver. Further investigation of these results is warranted.

In addition to gene methylation modification, lncRNAs also have an effect on physiological processes by regulation of gene expression and protein function [36]. Over 1000 lncRNAs have been reported to be associated with fatty liver [23]. Our results identified 67 differentially expressed lncRNAs, only two of which were annotated. A study of a NAFLD mouse model induced by an HFD showed over 290 lncRNAs to be differentially expressed [22]. *PER2*, a regulator of circadian rhythm, was positively regulated by lncRNA FLRL6 [22], which indicated that a regulatory relationship may be important in NAFLD progression because PER2 has an effect on hepatic lipid metabolism through PPARγ [37]. In this study, *PER2* was positively regulated by LNC_010240. We, therefore, inferred that LNC_010240 had a similar function to lncRNA FLRL6 in liver lipid metabolism. Although a large number of lncRNAs were detected, their regulatory mechanisms remain largely unknown [38]. Guo et al. classified lncRNAs to four classes and found the first class to respond to HFD and the third class to respond to liver or metabolic disease [39]. We also divided lncRNAs into thirteen clusters based on the expression pattern that contributed to the function. We found the lncRNA of cluster I to be mainly annotated to lipid metabolism pathways, especially LNC_006756, LNC_012355, and LNC_005024, whose target genes are involved in liver lipogenesis and transport. The lncRNAs of cluster IV and X were annotated to immune-related pathways, including LNC_010111, LNC_010862, and LNC_001272. These results provide candidate lncRNAs that are associated with chicken fatty liver. Besides that, it has been reported that lncRNA has a regulation effect on target genes by the cis way [40]. We found that LNC_008671, LNC_009039, and LNC_010494 had over 10 differentially expressed *cis* target genes, which suggested that these lncRNAs had a pivotal role in the process of fatty liver generation by regulation of gene expression.

Increasing evidence suggests potential crosstalk between DNA methylation and lncRNA interaction networks [41,42]. We integrated the methylation and lncRNA profile to detect common target genes. For target genes of lncRNAs, we found 23 genes (e.g., *ABCD3*, *BLMH*, and *HAO1*) that may be positively or negatively regulated by DNA methylation and may underlie the regulation of chicken fatty liver. These results provided powerful evidence that epigenetic changes play a critical role in the development of chicken fatty liver by regulation of gene expression. ABCD3 is a transporter of very-long-chain fatty acids that cause lipid accumulation in peroxisomes [43]. Overexpression of *ABCD3* results in β-oxidation of palmitic acid [44]. The regulation of ABCD3 is an important target of lipid transport and metabolism. Currently, no studies have explored epigenetic modifications of *ABCD3*. We found methylation of *ABCD3* to be associated with transcriptional repression and that the expression of *ABCD3* may be regulated by LNC_012355. These results provided a candidate lncRNA and methylation marker for a new regulatory mechanism of abnormal lipid metabolism. HAO1, a liver-specific peroxisomal enzyme with high fatty acid oxidase activity, is targeted to peroxisomes. Abnormal alterations of this gene are connected to hepatic steatosis [45,46,47]. We detected a DMR in the gene body of *HAO1* and a candidate LNC_006765. But a clear association of epigenetic modification and transcription of *HAO1* requires further investigation. Both ABCD3 and HAO1 are associated with peroxisome, which indicates the peroxisome maybe a central location in the process of cellular lipid homeostasis. BLMH is an aminohydrolase and is related to some liver diseases. It has been reported that BLMH can interact with lipoprotein and play an important role in fatty liver via homocysteine metabolism [48]. Besides, *BLMH* is also correlated with hepatocellular carcinoma [49]. However, no studies focused on the epigenetic modification of this gene. In this study, we discovered a candidate lncRNA and increased methylation level on the gene body of this gene, which provided a new idea to explore the regulatory mechanism of this gene. In addition, many other genes were also found to be regulated by epigenetic factors and related to liver disease, such as *MARCH1* [50,51], *LIMD2* [52], *SLC39A8* [52], etc. Meanwhile, some genes have not been proved to be included in the process of fatty liver or other liver disease, such as *ASPA*, *CCDC18*, etc. Which provided new targets for the epigenetic study of fatty liver. 

In conclusion, we have provided a comprehensive epigenetic and transcriptomic profile for chicken FLS. Besides, the targets (e.g., *HAO1*, *ABCD3*, and *BLMH*) and epigenetic markers identified by the integration analysis could contribute to the understanding of the molecular mechanism of FLS, and perhaps indicate a new research focus of chicken FLS.

## 4. Materials and Methods

### 4.1. Ethics Statement

All the animal experiments in this study were conducted in accordance with the guidelines for experimental animals established by the Ministry of Science and Technology (Beijing, China). Ethical approval on animal survival was given by the Science Research Department (responsible for animal welfare) of the Institute of Animal Sciences (IAS, Beijing, China), the Chinese Academy of Agricultural Sciences (CAAS, Beijing, China) with the following reference number: IASCAAS-AE-03.

### 4.2. Animal Model and Environment

One dwarf chicken line of JXH chicken, highly susceptible to fatty liver, was obtained from the Institute of Animal Sciences, Chinese Academy of Agricultural Sciences. These chickens were evaluated, as reported by Zhang [10]. One fatty liver group and one control group were generated from this line. Briefly, the initial JXH chickens (F0 generation) were randomly assigned to two groups and were fed HFD (fatty liver group) and basal diet (control diet), respectively. The offsprings (F1-F3) were produced by male chickens with (fatty liver group) or without (control group) fatty liver. All the offsprings (F1-F3) in two groups were fed a basal diet. For this study, male chickens from the F3 generation of the fatty liver group (*n* = 70) and the control group (*n* = 52) (Figure S3) were used. All chickens were fed the same basal diet formulated according to NRC (1994) and NY/T (33-2004), and raised in three-story step cages (one chicken per cage) under the recommended environmental conditions. Feed and water were provided ad libitum during the study.

### 4.3. Sample Collection

Blood samples were collected before the tissue sample collection. The serum was isolated after incubation for 1 h at 37 °C and stored at −80 °C. In the 42nd week after hatching, all the male chickens were euthanized by carbon dioxide anesthesia and exsanguination by severing the carotid artery after 12-h fasting. The traits (BW, DW, EW, AFW, and LW) were recorded from part of chickens (two out of five chickens at random). The liver was removed and collected. A portion of the liver was snap-frozen and stored at −80 °C for future RNA and methylation analysis, another portion of the liver was fixed in 4% paraformaldehyde for histological analysis.

### 4.4. Serum Biochemical Analysis and Liver Histology

The concentration of TG, TC, HDL, and LDL was tested with colorimetric reagents (Nanjing Jiancheng Bioengineering Institute, Nanjing, China). The paraformaldehyde-fixed liver was sectioned and stained with H&E or oil red O (Beijing Xuebang Science and Technology Co., Ltd., Beijing, China).

### 4.5. Evaluation of fatty liver

Fatty liver was assessed by pathological examination of liver H&E and oil red O stained sections [10]. Fatty liver was identified when the extent of fatty degeneration was greater than 33%, as well as excessive deposition of lipid in hepatocyte. The liver phenotype was also assessed.

### 4.6. Sequencing and Identification of Differentially Expressed LncRNAs and mRNAs

Total RNA was isolated from livers of male chickens with (*n* = 3, four samples were used to sequencing but one failed) and without (*n* = 4) fatty liver and assessed by agarose gel electrophoresis (Figure S4). Complementary DNA (cDNA) libraries were prepared using a NEBNext^®^ Ultra™ Directional RNA Library Prep Kit for Illumina^®^ (Ipswich, MA, USA). After removing rRNA, 150 bp paired-end reads were generated with the Illumina HiSeq X Ten platform (Novogene, Beijing, China). High-quality clean reads were obtained by the removal of low-quality reads from raw data with an in-hour script. An index reference genome (Gallus 5.0) was built and clean reads were aligned to the reference genome using HISAT2 v2.0.4 (https://ccb.jhu.edu/software/hisat2/index.shtml) [53]. Mapped reads were assembled by StringTie (http://ccb.jhu.edu/software/stringtie/) [54]. The expression normalization was performed by DESeq2 (https://bioconductor.org/packages/release/bioc/html/DESeq2.html) [55], and DEGs were defined as |FC| > 1.5 with *p* < 0.05. We identified lncRNA as previously described [56] using coding potential analysis with CNCI, CPC, and PFAM [57,58,59]. Differentially expressed lncRNAs were defined with DESeq2 (adjust *p*-value < 0.05, |FC| > 1.5).

All DEGs were annotated from the Ensembl database (http://asia.ensembl.org/index.html) [60], and a statistic for lipid- and glucose-related DEGs was conducted based on their biological process, as well as immune-related DEGs.

### 4.7. Quantitative Real-Time PCR

Total RNA from 26 frozen liver tissues (12 from the fatty liver group and 14 from the control group) was obtained using an RNA Isolation Kit (Tiangen, Beijing, China). The mRNA was converted to cDNA with a FastQuant RT Kit (Tiangen) following the manufacturer’s instructions. Primers of selected genes were designed based on chicken coding region sequences from the Ensembl database, which are shown in Table 3. *ACTB* and *RPS6* were selected as reference genes for normalization. The qRT-PCR was conducted in triplicate with the SYBR Premix Ex TaqTM reagent Kit (TAKARA, Kusatsu, Japan) with the QuantStuio 7 Flex Real-Time PCR System (Waltham, MA, USA), with the following program: 95 °C for 3 min, 40 cycles of 95 °C for 3 s, annealing temperature for 34 s. Results were analyzed by the 2^−^^ΔΔ*C*t^ method [61]. The correlation coefficient (R^2^) between qRT-PCR and RNA-seq was acquired from Pearson analysis.

### 4.8. Construction and Analysis of lncRNA-mRNA Network

Pearson correlation analysis of lncRNAs was performed, from which thirteen clusters were classified by hierarchical clustering methods [39], with the Pearson correlation coefficient (PCC) > 0.7 between any two lncRNAs in one cluster. Then the *cis* and *trans* target genes of differentially expressed lncRNAs were predicted. For *trans* target genes, we calculated the PCC and significant *p*-value for the expression levels of each lncRNA-mRNA pair. A lncRNA-mRNA network was constructed with the trans target genes (*p* < 0.01, |PCC| > 0.95) using Cytoscape software [62]. Gene Ontology annotation was applied with KOBAS (http://kobas.cbi.pku.edu.cn/) [63] to elucidate the function of each cluster of differentially expressed lncRNAs. For *cis* target genes, we identified chromosomal co-expressed genes within 300 kbps upstream and downstream of differentially expressed lncRNAs.

### 4.9. Whole-Genome Bisulfite Sequencing and DMGs Identification

Genomic DNA was obtained from the same liver samples (*n* = 3 in the fatty liver group and *n* = 4 in the control group) as used for mRNA sequencing. It was assessed by agarose gel electrophoresis (Figure S5). Library preparation was performed as previously described [64] and sequenced with the Illumina HiSeq X Ten platform (Novogene, Beijing, China). Subsequently, 150 bp paired-end reads were generated. The quality of raw data was assessed by FastQC. Clean data were filtered with specific conditions, as previously reported [65]. Before analysis, the transformed chicken reference genome was bisulfite-converted (C to T and G to A). Clean reads were fully bisulfite-converted (C to T and G to A) and mapped to the converted genome version using Bismark [66].

Methylation level (ML) for each C site was analyzed by the sliding-window approach with the sum of methylated and unmethylated reads counted, where: ML (C) = reads (mC) / (reads (mC) + reads (umC)). Herein the focus was on the methylation level for each CpG site. DMRs were identified using DSS software [67,68,69]. Spatial correlation and biological replicates were both considered in the detection process. DMGs were defined as the gene body and promoter region that overlapped with DMRs.

Pearson correlation analysis was used to assess the overlapped genes of DMGs and DEGs. A *p*-value of 0.05 was set as the threshold for significant correlation. Genes with a *p*-value of 0.1 were also taken into consideration.

### 4.10. KEGG Pathways Analysis

Pathway enrichment analysis was performed with ClusterProfiler [70] to explore the function of DEGs and DMGs, respectively. A *p*-value of 0.05 was set as the threshold for significant enrichment.

### 4.11. Statistical Analysis

SPSS 25.0 (SPSS, Chicago, IL, USA) was used for statistical analysis. Data are shown as mean ± standard error. Comparisons were performed by Student’s *t*-test or Wilcoxon signed-rank test. A *p*-value < 0.05 (*) and *p*-value < 0.01 (**) implied a statistically significant difference and highly significant difference, respectively. Graphics were drawn using GraphPad Prism 7 (GraphPad Software, San Diego, CA, USA).

## Figures and Tables

**Table 1:** Pathways enriched by both differentially expressed genes (DEGs) and differentially methylated genes (DMGs).

ID	Pathway	Tendency of DEG	*p*-Value ^1^	DMR	*p*-Value ^2^
gga01200	Carbon metabolism	up	8.53 × 10^−4^	promoter	3.69 × 10^−2^
gga00020	Citrate cycle (TCA cycle)	up	1.70 × 10^−2^	promoter	4.82 ×10^−2^
gga04115	p53 signaling pathway	up	2.88 ×10^−2^	gene body	3.76 × 10^−2^
gga04020	Calcium signaling pathway	down	8.94 × 10^−3^	gene body	1.35 × 10^−2^
gga04933	AGE-RAGE signaling pathway in diabetic complications	down	4.41 × 10^−2^	gene body	3.09 × 10^−2^

^1^*p*-value of enrichment analysis with DEGs; ^2^*p*-value of enrichment analysis with DMGs.

**Table 2:** Target genes regulated by both long noncoding RNA (lncRNA) and DNA methylation.

lncRNA	Regulation	Gene	Log2FC	DMR	Methylation Difference
LNC_008609, LNC_008671	*trans*	*LIMD2*	−0.83	gene body	−0.39
LNC_012679	*trans*	*BLMH* ^1^	0.67	gene body	0.35
LNC_008303	*trans*	*ASPA*	1.41	promoter	−0.16
LNC_012355	*trans, cis*	*ABCD3* ^2^	1.05	gene body	−0.49
LNC_010111	*trans, cis*	*CCDC18*	−0.74	gene body	0.35
LNC_006756	*trans*	*HAO1* ^2^	0.99	gene body	0.33
LNC_010111, LNC_010862	*trans*	*FLVCR2*	−1.16	gene body	−0.4
LNC_010073, LNC_010240	*trans*	*FAM13A*	0.89	gene body	−0.18
LNC_002556	*trans, cis*	*ENSGALG00000010639*	−0.73	gene body	0.52
LNC_009039	*trans, cis*	*ENSGALG00000011528*	−0.91	gene body	0.6
LNC_000820	*trans*	*SLC39A8*	0.73	gene body	−0.2
LNC_010111	*trans*	*MYO16*	1.44	gene body	−0.32
LNC_000333	*trans*	*COTL1*	−0.84	gene body	−0.36
LNC_007320, LNC_007320	*trans*	*CELF2*	−0.72	gene body	−0.24, −0.31
LNC_005357, LNC_007350, LNC_010111, LNC_010862	*trans*	*RAC2* ^1^	−0.78	gene body	−0.17
LNC_001439, LNC_001531, LNC_007015, LNC_010098	*trans*	*JAM2*	0.59	gene body	0.38
LNC_001714, LNC_001742, LNC_006829, LNC_012722	*trans*	*WDPCP*	1.92	gene body	−0.24
LNC_001439, LNC_001531, LNC_005357, LNC_007015, LNC_010098, LNC_010111	*trans*	*ENSGALG00000033919*	0.84	gene body	0.11
LNC_001272, LNC_002705, LNC_003079, LNC_007151, LNC_010862, LNC_011070	*trans*	*DOCK2* ^1^	−0.62	gene body	0.19
LNC_002705, LNC_003079, LNC_008608, LNC_012083, LNC_012722	*trans*	*DIP2C*	0.92	gene body	−0.22
LNC_001272, LNC_002705, LNC_003079, LNC_007151, LNC_008608, LNC_010862, LNC_011070	*trans*	*GALNT17*	−1.32	gene body	−0.18
ENSGALT00000085791, LNC_001272, LNC_007151, LNC_007350, LNC_010862, LNC_011070	*trans*	*MARCH1* ^1^	−0.78	gene body	−0.27, −0.33
LNC_001272, LNC_001439, LNC_002556, LNC_005357, LNC_007151, LNC_007350, LNC_008609, LNC_010111, LNC_010494	*trans*	*MEGF11*	3.15	gene body	−0.17

^1^ Immune-related genes; ^2^ Lipid- and glucose-related genes.

**Table 3:** Specific primers for qPCR.

Gene ID	Gene	Primer Sequence	Product Size (bp)
ENSGALG00000002549	*RGS1*	F:5′-AGGATTTACGAGGAGTTTGT-3′	105
		R:5′-TGTGTGAGTTGGGTCTTG-3′	
ENSGALG00000033511	*CYP8B1*	F:5′-GGATAAGTGAACAAGACCAGTA-3′	132
		R:5′-GATACAAGAGGAGCCAGAAG-3′	
ENSGALG00000007904	*DLAT*	F:5′-TTGCTCTCCCTGCTCTGT-3′	127
		R:5′-CCTATTGTGGCTTTATCTGTCT-3′	
ENSGALG00000013594	*PARD6G*	F:5′-GCCAACAGCCATAACCTT-3′	184
		R:5′-CCTCTTCGTCACTCTCCA-3′	
ENSGALG00000005739	*SCD*	F:5′-GGCTGACAAAGTGGTGATG-3′	137
		R:5′-GGATGGCTGGAATGAAGA-3′	
ENSGALG00000007178	*FADS2*	F:5′-CTGAGGAAGACAGCAGAGGACAT-3′	153
		R:5′-GCAGGCAAGGATTAGAGTTGTG-3′	
ENSGALG00000025796	*ADI1*	F:5′-ACATGGACGAGTCCCAGGAG-3′	113
		R:5′-AGCATCCAATCTGCGGTAGG-3′	
ENSGALG00000011016	*PGM1*	F:5′-ACGGTGAAAACCAAGGCGT-3′	103
		R:5′-TGAAGTTCTCGGCGTAGTGG-3′	
ENSGALG00000023395	*PLIN1*	F:5′-GCAATCCAGGGCTTACAG-3′	171
		R:5′-ATCCAGACGACCAGTTCC-3′	
ENSGALG00000008845	*HAO1*	F:5′-CGGTTTGTGTTGCTGATTT-3′	116
		R:5′-TGCTGCTACATTATCTGCTA-3′	
ENSGALG00000015082	*RPS6*	F:5′-GAGCGCAACGTGAGAACATT-3′	92
		R:5′-CGGACAACATAGCCCTTCCA-3′	
ENSGALG00000009621	*ACTB*	F:5′-GAGAAATTGTGCGTGACATCA-3′	152
		R:5′-CCTGAACCTCTCATTGCCA-3′	

F: forward, R: reverse.

## Supplementary Materials

The following are available online at https://www.mdpi.com/1422-0067/21/5/1800/s1.

## Abbreviations

DEG	Differentially Expressed Gene
DMG	Differentially Methylated Gene
DMR	Differentially Methylated Region
FC	Fold Change
FLS	Fatty Liver Syndrome
HFD	High Fat Diet
LncRNA	Long Noncoding RNA
NAFLD	Non-Alcoholic Fatty Liver Disease
PCC	Pearson Correlation Coefficient
HDL	High-Density Lipoprotein
LDL	Low-Density lipoprotein
TC	Total Cholesterol
TG	Triglyceride
